# Factors affecting the effectiveness of captive-bolt stunning of reindeer (*Rangifer tarandus tarandus* L.) at commercial slaughter in Sweden

**DOI:** 10.1186/s13028-026-00852-x

**Published:** 2026-01-28

**Authors:** Arja Helena Kautto, Margareta Steen, Ivar Vågsholm, Charlotte Berg

**Affiliations:** 1 Department of Animal Biosciences, Swedish University of Agricultural Sciences, Ulls Väg 26, 751 23 Uppsala, Sweden; 2 Control Support Unit, Department of Food Control, Dag Hammarskjöldsvägen 56 A, 751 26 Uppsala, Sweden; 3 Swedish Centre for Animal Welfare (SCAW), Swedish University of Agricultural Sciences, Ulls Väg 26, Uppsala, Sweden; 4 Department of Wildlife, Fish, and Environmental Studies, Swedish University of Agricultural Sciences, 901 83 Umeå, Sweden; 5 Department of Applied Animal Science and Welfare, Swedish University of Agricultural Sciences, Gråbrödragatan 19, 531 31 Skara, Sweden

**Keywords:** Animal welfare, Bleeding, Cervid, Control development, Farmed game, Official controls, Slaughter, Stun-to-stick interval

## Abstract

**Background:**

Slaughterhouses for all animals, including reindeer, must achieve the goal of high animal welfare. They must carry out regular checks on stunning effectiveness and key parameters to ensure that animals do not display any signs of consciousness, and display the expected signs of unconsciousness, in the period between stunning and death. The official control verifying this performance must be based on scientific evidence. Experience gained and scientific development are to be considered when control and regulations are developed. The purpose of this study was to evaluate penetrative captive bolt-stunning quality in stunning of reindeer (*Rangifer tarandus tarandus* L.). We investigated stun-to stick interval in relation to indicators of consciousness and unconsciousness as well as factors affecting the time between stunning and sticking to generate evidence-based knowledge for optimising animal welfare by best practise at stunning. Stun-to-stick interval was measured for 1,590 reindeer during eight slaughter days at two abattoirs during slaughter season 2015–2016. The variables recorded were abattoir (AA, AB), season, type of stunning (cartridge-powered/ pneumatic captive bolt gun), level of experience of the stunning operator (one/three/five years), animal category (calf/male/female), and origin of the reindeer (mountain/forest), number of stunning attempts per reindeer, indicators of possible remaining or regaining of consciousness and indicators of unconsciousness until the end of bleeding.

**Results:**

Mean stun-to-stick interval for effective stuns was 44.1 s (95% confidence interval (CI 95%) = 43.6–44.6). Longer stunning experience was associated with shorter mean stun-to-stick interval (P < 0.001) as well as a quicker slaughter hoisting process in one of the two abattoirs (P = 0.016). In 5.3% (83/1,569) of one-shot stuns, stun-to-stick interval exceeded 60 s, where stunning operators with short experience had significantly more cases than more experienced operators (P < 0.001). Ineffective stuns (> one shot/reindeer), with continued standing posture of a reindeer post-shot, comprised 1.3% (21/1590) of all stuns with significant relationship to adult male reindeer and none to stunning operator experience.

**Conclusions:**

Proper standard operating procedures including maintain of equipment as well as a supervised training period for operators as suitable risk management activities are recommended. Official controls could be most effective by focusing on these factors.

**Supplementary Information:**

The online version contains supplementary material available at 10.1186/s13028-026-00852-x.

## Background

In response to societal demands, the slaughter industries focus on continuous improvement in meat processing operations to optimise animal welfare as well as meat quality. The Terrestrial Animal Health Code guidelines from World Organization for Animal Health (WOAH) include recommendations concerning the handling, restraining, stunning and bleeding of animals at slaughterhouses [1]. The regulations in the European Union (EU) place responsibility on food business operators (FBOs) for food safety and animal welfare [2–5] and the competent authorities (CA) [6–8] for verification of FBOs’ compliance with these regulations. Sweden has specific stricter national regulations and guidelines on the slaughter and killing of animals, including reindeer (*Rangifer tarandus tarandus* L.) issued by the competent authority (CA) Swedish Board of Agriculture [9]. The FBOs’ development of standard operating procedures (SOPs) and CA’s control instructions are partially based on the European Food Safety Authority (EFSA) guidelines [10, 11]. In commercial slaughter there is consensus that handling, stunning and bleeding at slaughter can be performed with a high level of animal welfare [12, 13].

**Fig. 1:**
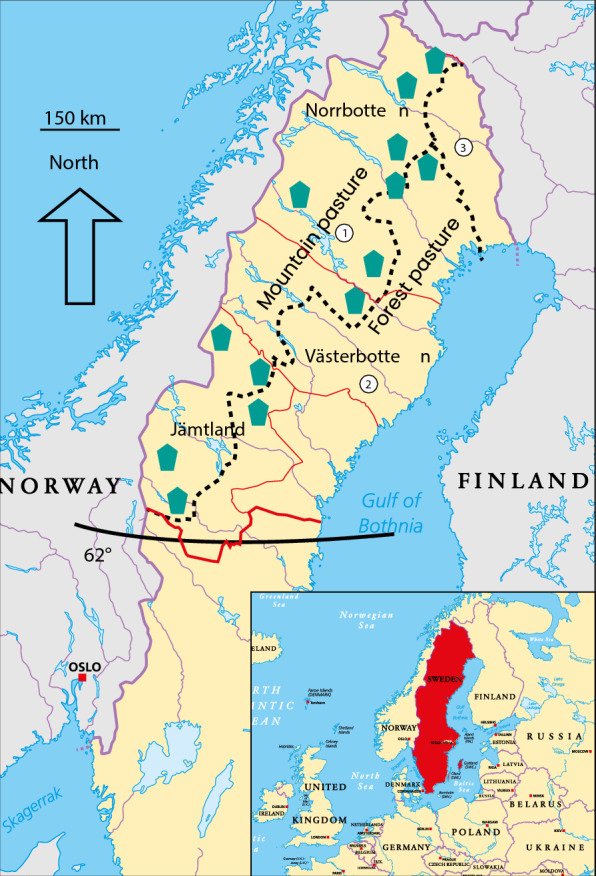
Reindeer herding area and reindeer abattoirs in Sweden. Reindeer abattoirs = green dots, 1 = summer pastures, 2 = winter pastures, 3 = Torne Valley are separated by a dotted line. Latitude 62° and southern border for the reindeer herding area indicated. Norrbotten, Västerbotten and Jämtland are the administrative county units

Around 250,000 reindeer [14] graze freely and are managed by owners on natural pastures across Northern Sweden that occupy around 50% (200,000 km^2^) of Sweden’s total terrestrial area (Fig. 1). Reindeer are gathered by their owners during the slaughter season (September–April) in specific fenced areas on the pastureland for slaughter selection. The specific vehicles used for transport to the reindeer abattoirs are approved by the county veterinary officers. Transports are regulated regarding grouping, size of the boxes and transport time [15]. After transport, reindeer are unloaded into fenced outdoor lairage areas and slaughtered as soon as possible, normally on the same day or in the morning of the following day. Management practises are traditional and in line with the results from studies on deer management in New Zealand [16]. For the past 10 years, 40,000–60,000 reindeer have been commercially slaughtered during each year [14] at abattoirs with outdoor lairage areas surrounded by fences approved by the competent authority Swedish Food Agency (SFA), in accordance with EU legislation [3, 4]. These 13 abattoirs are located along the reindeer herding area in Northern Sweden (Fig. 1).

Ante mortem inspection and food chain information checks are performed by the official veterinarian (OV) from the SFA. Stun boxes are designed to accommodate adult reindeer at least in groups of two or three, to reduce the level of stress and leave less room for the reindeer to move around during restraint. The stunning operator is allowed to manually restrain the reindeer by holding it by the antlers if these are not in velvet [9]. The captive bolt gun (CBG) is used for stunning reindeer in Finland, Norway and Sweden. In cases of slaughter for household consumption, weapons with a free bullet (at least 3.2 g and 800 J E100) may be used at maximum range of 5 m from the reindeer [9].

After stunning consecutively all reindeer in the box and before hoisting, the stunning operator routinely checks every reindeer for absence of signs of consciousness and presence of signs of unconsciousness. Reindeer are secured by one hind leg below the tarsus, hooked onto a rail and hydraulically hoisted onto the processing line, head down. Animal welfare indicators are verified by the OV in accordance with a specific control plan or ad-hoc in the event of problems.

Exsanguination should commence as soon as possible after stunning [4] and the two carotid arteries, or the vessels from which they arise, must be systematically severed [5]. According to Swedish and international [1] guidelines, the stun-to-stick interval should be kept below 60 s [17]. The animals are checked for relevant signs of consciousness/unconsciousness also after sticking, and carcasses should only be processed once absence of signs of life has been verified [5]. There is no agreed formal definition of death in animals comparable to that established for humans in Swedish law [18]. The death of an animal is verified by the absence of signs of life, such as breathing, brainstem reflexes and (in case of doubt based on other indicators) heartbeats, and by assessment of sufficient bleeding [19]. Moreover, there are indicators of death, such as rigor mortis, which cannot directly be used in slaughter context on-line. Postmortem inspection is performed on the carcass and organs, and the carcass health marked accordingly, if it is considered fit for human consumption [8].

Proper maintenance of weapons, signs of wear to the bolt and barrel, cartridge storage and well-trained personnel are included in the SOPs for FBOs and verified by the Competent Authority (CA). The Swedish Board of Agriculture (SBA), as central competent authority (CCA) for the animal welfare legislation, stipulates that back-up stunning devices must be kept at abattoirs for stunning both domestic and farmed game mammals. All personnel working with live animals at slaughterhouses must hold a certificate of competence guaranteeing that they meet the requirements to effectively stun different species of animals at slaughter [5]. The stunning operators are not allowed to work before training is completed and a certificate of competence has been issued. The CA must verify this requirement during planned or ad-hoc checks [8].

The purpose of stunning is to induce unconsciousness and insensibility to external stimuli that might otherwise generate feelings of pain, fear or stress. This state shall persist until death [20]. Captive bolt induced injury to the central nervous system, focusing on the thalamus, the hypothalamus, midbrain and rostral pons, leads to impaired consciousness, as such injuries prohibit stimulation of the cortex and without such stimulation the animal is unconscious [21]. Several different indicators, emanating from various parts of the brain, including the medulla and the brain stem, may be used for verifying a successful stun [21]. Some of these indicators (e.g. loss of posture, loss of corneal reflex, absence of rhythmic breathing) indicate unconsciousness, while others indicate a risk of (return to) consciousness, i.e. a failed stun. The FBO must carry out regular checks on stunning effectiveness and key parameters to ensure that animals do not display any signs of consciousness or sensibility in the period between stunning and death [5]. To achieve the goal of high animal welfare, the SOPs and official checks must verify this performance. The official control must be based on principles of risk analysis consisting of risk assessment and risk management based on scientific evidence to be able to reach a proportional and non-restrictive approach [2]. Experience gained by competent authorities and FBOs as well as scientific development are needed and to be considered when control and regulations are developed [6].

**Fig. 2:**
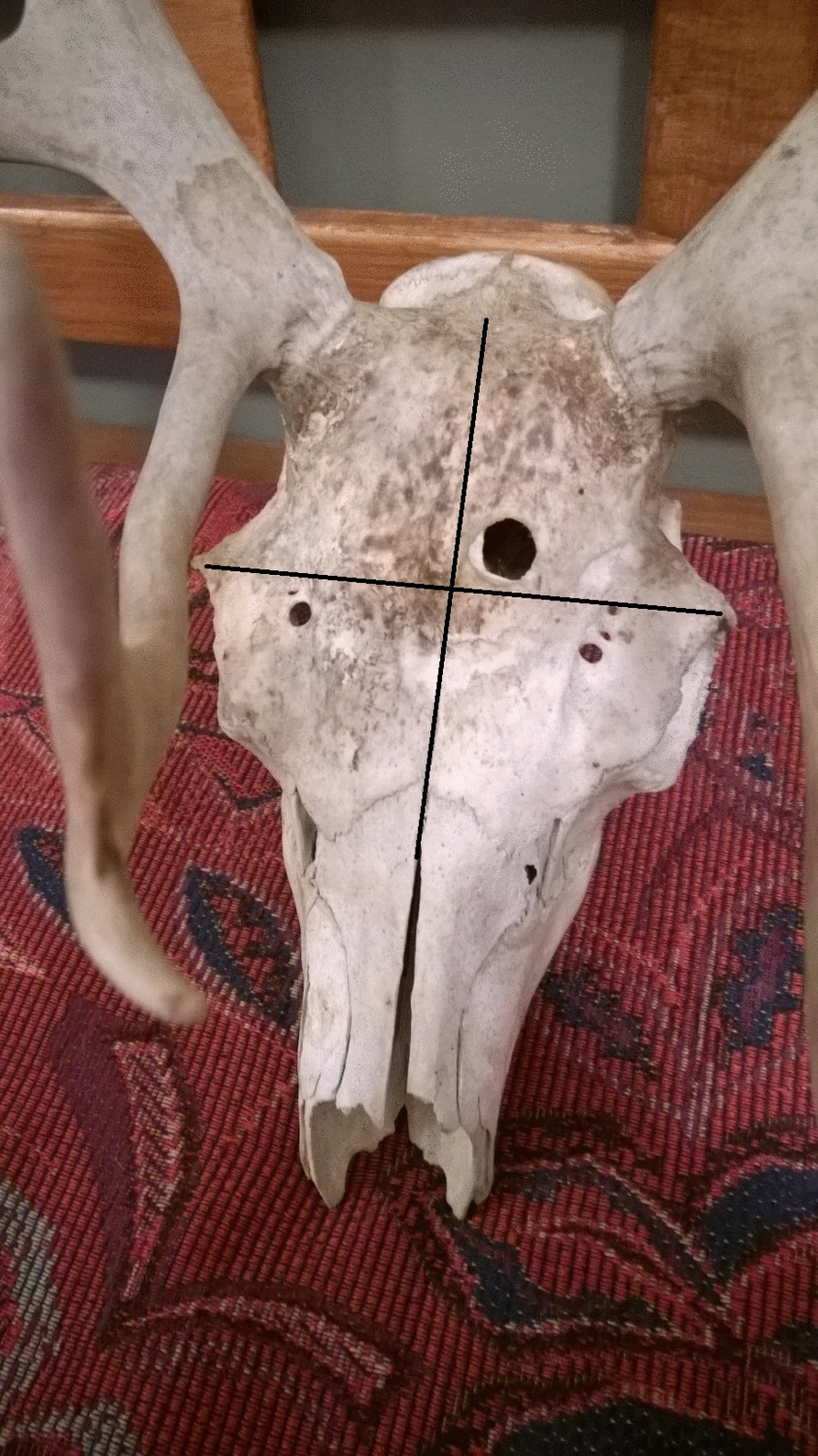
Stunning point in the adult female reindeer cranium. Crossing lines indicating the approved position according to national guidelines. The sagittal shot hole is not exactly according to the guidelines but the stun of this reindeer in normal slaughter was effective. This individual was not part of the study

The method applied for stunning of reindeer is induced damage of brain tissue and extensive haemorrhage to the target zone using a captive bolt into the shot point of the forehead, known as the frontal shooting position (Fig. 2). CBGs are designed to fire a retractable steel bolt into the head of reindeer standing in a specially designed stunning box. Unconsciousness is caused by a direct damage of the brain when the bolt is delivered in right orientation to the animal’s brain [22] and by the arterial ruptures leading to severe haemorrhages affecting several regions of the central nervous system [23]. The bolt must be of sufficient length and the kinetic energy great enough to reach the target zone and cause unconsciousness of the animal [24]. Peak bolt velocity and kinetic energy in pneumatically powered CBG are related to the airline pressure of the system and must be sufficiently high to achieve effective stunning quality [25]. When a cartridge-powered CBG is to be used, the choice of weapon should take the type of cartridges, the kinetic energy delivered to the head of the animal, and the bolt penetration width and depth into consideration in relation to the species and animal type in question [22]. Moreover, correct maintenance of the technical devices used is the most important factor for inhibiting return to sensibility following the stun, particularly when using a cartridge-powered device [23, 26, 27]. Seven of the 13 approved reindeer abattoirs in Sweden today use pneumatically powered CBGs, while the remaining six use cartridge-powered CBGs as their primary method of stunning and all 13 abattoirs have a cartridge-powered CBG as back-up (Loran Postolovski, pers. comm. 2025).

There is a lack of information on captive-bolt stunning in reindeer concerning the stun-to-stick intervals in relation to various indicators of consciousness and unconsciousness as well as factors affecting the time between stunning and sticking, i.e. start of bleeding. The aim of the present study was to provide new knowledge in this area.

Specific objectives of the study were to:

- Evaluate captive bolt stunning effectiveness at commercial slaughter of reindeer by defined indicators,
- Identify the indicators the reindeer display at effective versus ineffective stunning,
- Produce scientific evidence regarding the existing SOP recommendations of stun-to-stick intervals and possible factors that affect this time and
- Give evidence-based recommendations to FBOs improving SOPs and to CA developing effective risk based official controls.

## Methods

### Study population and study unit

The reindeer, in total 1,590 animals, originated from four different pasture areas. After direct transport, maximum eight hours without stop, and unloading into large outdoor enclosures adjacent to the abattoirs, the animals were slaughtered within 12 h after arrival. Eight separate slaughter days were randomly selected during the period September 2015 to April 2016. The stunning was performed according to the standard operational procedures at the abattoirs in question. All reindeer in the stunning box were first shot consecutively with short intervals, and the indicators were checked for all of them before hoisting. Reindeer were in the narrow stainless-steel stunning box with concrete floor in small groups; two at the time for males, three for females or calves. The stunning operator was standing alone beside the box, slightly above the reindeer, and waiting until the reindeer stands still. No further restraint, except the possibility to hold the animal by one antler, is used for reindeer at commercial slaughter. These two abattoirs are approved for the slaughter of reindeer and the size of stunning box in the slaughter line is specific for this slaughter. The hoisting system at abattoir AA was older and slower than at abattoir AB.

The individual reindeer was the study unit. An experienced veterinarian (“observer”) monitored all reindeer at stunning and using Maxfit® timers (access to three timers) to measure the stun-to-stick interval. The timer was activated by the observer when the shot hit the reindeer skull and stopped when the knife was stuck into the arterial bifurcation in the thorax foramen. The results were documented directly on a spread sheet and later digitalised in an Excel file.

### Stunning method

The cartridge-powered method used at both participating abattoirs was a standard CBG weapon (CASH® Special) with pistol grip and powder-loaded cartridges (CASH® cartridge 0.25 Blue 3G). The pneumatic method used in both abattoirs was a standard pneumatic CBG (EFA VB 215 penetrating stunner) with pistol grip connected to an air compressor via an air hose equipped with an air-shock eliminator and a pressure regulator. The operating airline pressure was 12 bar and air consumption per shot 12 L in the pneumatic system with maximum penetration depth 101 mm.

### Study design

This was an observational study with time to sticking (start of bleeding, exsanguination) as endpoint and evaluation of possible factors influencing the stun-to-stick interval. Documented baseline information consisted of: identification of the abattoir (AA, AB), season (Autumn, Winter, Spring), stunning operator (persons A-E), stunning operator experience level (operator E one year, A and C three years, B and D five years), type of penetrating CBG stunning equipment (cartridge-powered or pneumatic), animal category (calf, adult male, adult female), and origin of the reindeer (main grazing area, i.e., mountain or forest). The sex of calves under one year old was not recorded. Stunning operators A and B operated in abattoir AA, C and E in abattoir AB, and operator D both in AA and AB. The indicators checked by the observer at different stages of the killing process are presented in Table 1. Ineffective stuns that resulted in any kind of observed signs of consciousness according to Verhoeven et al. [20] resulted in re-stunning until effective stun was achieved. This was a responsibility of the stunning operator. Any reindeer that were not rendered unconscious were stunned with a CBG again, after decision made by the stunning operator. The observer standing close to the stunning box and with good sight into it recorded own personal observations and the results from the corneal reflex tests and those of the stunning operator according to the list of indicators. Any oral communication between the observer and the stunning operator took place after bleeding of the reindeer in question.

**Table 1:** Indicators checked during and after stunning and bleeding in commercial slaughter, based on Verhoeven et al. [20]

Indicator	At stunning	After stunning	At bleeding	After bleeding
Hit point of bolt	X	X	X	X
Standing posture	X	X	*	*
Head righting	X	X	X	
Body righting	X	X	X	
Vocalization	X	X	X	
Rhythmic breathing		X	X	
Eye tracking		X	X	
Eye blinking		X	X	
Corneal reflex		X	X	X
Reaction on the sticking			X	

^*^standing posture not relevant because reindeer is hoisted and hanging on the rail

### Data processing

The three seasons were Autumn slaughter (September), before the rutting period for adult male reindeer; Winter slaughter (November-January) of calves and adults of both sexes; and Spring slaughter (March) of calves and adults of both sexes fed in corrals. Ineffective stuns were analysed separately from effective stuns. Effective stuns with stun-to-stick intervals exceeding 60 s were considered as delayed sticking based on the SBA recommendation at the national level. Ineffective stuns were those where more than one stun was needed to render the reindeer unconscious.

### Statistical analysis

Stun-to-stick interval for effective stuns, including delayed stickings, were analysed in terms of distribution (skewness and kurtosis), mean and standard error of the mean (SE) for the total data and in relation to the level of experience of the stunning operator. This was done for all the subsets of data (abattoirs AA and AB, experience levels; long, medium and short, stunning type cartridge and pneumatic, calf and adult, origin of reindeer, forest and mountain and seasons; autumn, winter and spring). Visualization was done as Kernel probability density violin plots of the data at different stun-to-stick times, median and quartiles for all stuns in relation to different variables [28].

Welch two sample t-test [29] was used to compare the means of the stun-to-stick intervals of effective stuns between abattoirs AA and AB, between the three different experience level of stunning operator, between stunning type cartridge and pneumatic, between animal type calf and adult, between origin of reindeer forest and mountain and between season autumn, winter and spring.

The input data, structured as a 2 × 2 matrix of frequencies, were analysed with a 95% confidence level and a two-sided alternative hypothesis. McNemar’s Chi-squared tests [30] were performed for effective stuns stun-to-stick interval (number of animals with a stun-to-stick interval > 60 s versus ≤ 60 s) and for different experience levels of the stunning operator as well as for differences in effective versus ineffective stunning between calves and adults, between reindeer males and females, between forest and mountain origin of reindeer and between winter and autumn. For results with low number of observations (expected less than 5 per cell) the Fishers Exact Test [31] was used.

Data were analysed using Software R® [32], with package “base” for the descriptive values, “datawizard” for kurtosis and skewness, “stats” for Welch Two Sample t-test and McNemar’s Chi-squared test, “ggplot” for visualisation. P-value < 0.05 was used to indicate significance.

## Results

### Descriptive results of stun-to-stick measurements

Most reindeer (n = 1,327, 85%) included in the analysis were slaughtered during the winter season and 61% (n = 975) were calves. A pneumatic CBG was used in 87% (n = 1,382) of stuns and a cartridge-powered CBG in 13% (n = 208). Abattoir AA slaughtered 37% (n = 590) and AB 63% (n = 1,000) of all reindeer in this study. Descriptive data on the number of reindeer (in total n = 1,590) in relation to different variables are presented in Table 2.

**Table 2:** Number of reindeer stunned during the study period September 2015-April 2016

Stunning operator/abattoir	Years	Animal type			Season			Method		Grazing area	
		All	Calves	Adults	A	W	S	CP	Pne	F	M
A/AA	3	345	255	90	0	345	0	1	344	39	306
B/AA	5	115	0	115	115	0	0	115	0	0	115
D/AA	5	130	76	54	0	0	130	13	117	24	106
Total /AA	-	590	331	259	115	345	130	129	461	63	527
C/AB D/AB E/AB Total/AB	3 5 1 -	326 461 213 1000	202 309 133 644	124 152 80 356	0 0 0 0	326 461 213 1000	0 0 0 0	3 66 10 79	323 395 203 921	238 148 139 525	88 313 74 475
Total AA + AB	-	1590	975	615	115	1345	130	208	1382	588	1002

AA and AB different abattoirs; A-E = five different stunning operators; 1,3,5 = years of experience; Autumn (A), Winter (W), Spring (S) = slaughter season; Cartridge-powered (CP), Pneumatic (Pne) CBG = method used for stunning; Forest (F), Mountain (M) = grazing area as proxy for origin of reindeer

The data of stun-to-stick interval were normally distributed showing low values of skewness (between -2 -and + 2) and kurtosis [33] in total and in all subsets. Only the data on stuns performed by stunning operators with long experience, had slightly higher kurtosis (7.539 ± 0.183) than the normally acceptable level of 7 [33], giving a slightly right-tailed distribution.

Mean stun-to-stick interval for effective stuns was 44.1 s (95% Confidence Interval (CI 95%) = 43.6–44.6). Stun-to-stick intervals for effective divided in animal type (calves, adults) in seconds, including 95% CI, and number and percentage of ineffective stuns combined and for abattoir AA and AB are presented in Table 3. The number and mean with CI 95% for stun-to-stick intervals for the origin of reindeer (forest, mountain) and season (autumn, spring, winter) are presented in Table 4.

**Table 3:** Stun-to-stick interval of reindeer at Abattoir AA, AB and combined (both)

Levels of experience*	Effective stuns Stun-to-stick interval, mean and standard error (SE) In seconds (CI 95%)			Number and per cent of ineffective stunned reindeer **					
	All	Calves	Adults	All	%		Calf		Adult
L/AA	41.8 (40.7–42.9)	40.6 (39.4–41.8)	40.0 (38.5–41.5)	3	-				3
M/AA	49.7 (48.6–50.8)	49.4 (48.3–50.5)	50.7 (47.9–53.5)	2	-		1		1
Tot/AA	46.5 (45.6–47.3)	47.4 (46.4–48.4)	45.3 (43.8–46.7)	5	0.9		1		4
L/AB	37.7 (37.2–38.2)	35.7 (35.2–36.2)	41.7 (40.6–42.8)	5	-		2		3
M/AB	45.5 (44.7–46.3)	43.0 (42.2–43.8)	49.5 (48.3–50.7)	4	-		0		4
S/AB	50.3 (48.9–51.7)	48.1 (46.6–49.6)	53.3 (51.1–55.5)	7	-		2		5
Tot/AB	42.9 (42.3–43.5)	40.6 (39.9–41.3)	47.2 (46.2–48.2)	16	1.6		4		12
L/both	39.0 (38.5–39.5)	36.7 (36.2–37.2)	42.1 (41.2–43.0)	8	1.1		2		6
M/both	47.6 (46.9–48.3)	46.5 (45.7–47.3)	49.9 (48.5–51.3)	6	0.1		1		5
S/both	50.0 (48.7–51.4)	48.1 (46.6–49.6)	53.3 (51.1–55.5)	7		3.3		2	5
Total	44.1 (43.6–44.6)	42.9 (42.3–43.5)	46.2 (45.4–47.0)	21		1.3		5	16

*Levels of experience of the stunning operator. L = long = five years, M = Medium = three years, S = short = one year

**Number of reindeer in stuns with more than one shot used (ineffective stun) recorded the same by both stunning operator and observer

**Table 4:** Stun-to-stick interval of reindeer for effective stuns.*

	Forest**	Mountain**	Autumn**	Winter**	Spring**
Mean (CI 95%)	45.5 (44.7–46.2)	43.4 (42.8 – 44.0)	42.0 (39.9–44.0)	44.6 (44.0–45.1)	41.8 (40.9–42.6)
Reindeer***	576	993	112	1327	130

*Mean and standard error (SE) in seconds with 95% confidence interval (CI 95%)

**Forest, Mountain = the origin of reindeer, Autumn, Winter, Spring = slaughter season

***Number of reindeer in each group

The mean stun-to-stick intervals with effective stuns were statistically different between stunning operator with long versus medium (P < 0.001), medium versus short (P < 0.001) and long versus short (P < 0.001) experience, between calf and adult reindeer (P < 0.001), between forest and mountain origin of reindeer (P = 0.009) and between abattoir AA and AB (P = 0.016), between season spring and winter (P < 0.001) and winter and autumn (P = 0.026). The mean stun-to-stick interval did not differ statistically between season spring and autumn (P = 0.262).

**Fig. 3:**
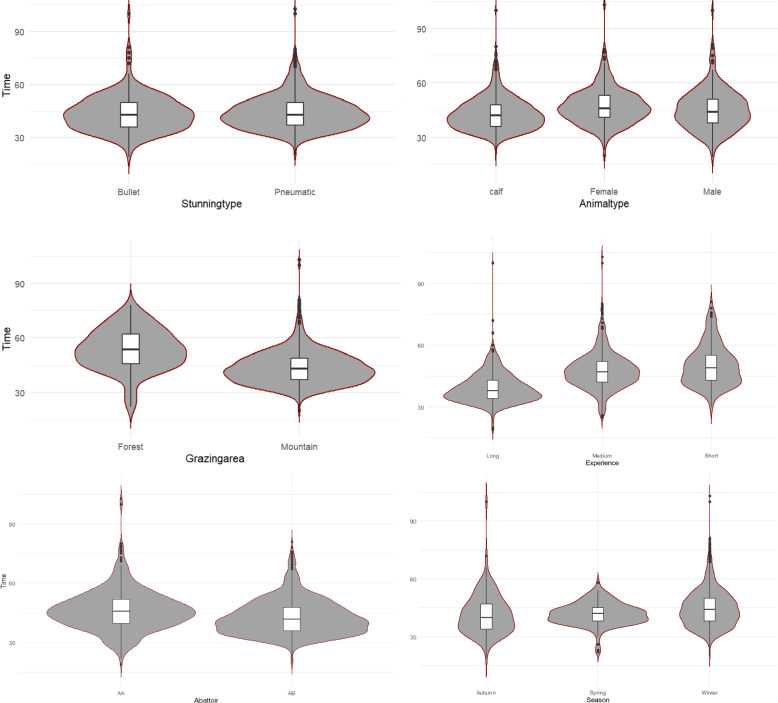
Probability density of the data at different stun-to-stick times for all stuns. Abattoir (AA versus AB), Experience (level of experience of the stunning operator as five years = long, three years = medium, one year = short), Grazing area (mountain/forest), Animal type (calf/male/female), Stunning type (“Bullet “ = cartridge-powered versus pneumatic powered captive bolt gun) and Season (Autumn, Spring, Winter). Box boundaries represent 25th (first quartile = Q1) and 75th (third quartile = Q3) percentiles, centre line shows median, whiskers minimum and maximum extend to a value within 1.5 × IQR (interquartile range), outliers are above Q3 and below Q1. Kernel probability density of data in relation to different variables used

The stun-to-stick interval data in relation to different variables are visualized in Fig. 3.

### Effective stun quality in relation to the 60-s recommendation

In total, 5.3% (83/1,569) of all effective stuns had a delayed sticking where the recommended limit of 60 s stun-to-stick interval was exceeded. Evaluations made by the stunning operator and observer concluded that reindeer with stun-to-stick intervals of > 60 s remained unconscious after stunning and sticking and during bleed out. There was statistically significant difference in number of > 60 s cases, with more experienced operators having the fewest, and less experienced operators having the most of the cases (Long versus Medium P < 0.001, Medium versus Short P = 0.008). Other variables showed non-significant differences in relation to exceeding 60 s stun-to-stick interval. The results structured as a 2 × 2 matrix of frequencies can be seen in supplemental material (S1).

Among ineffective stuns, a total time exceeding 60 s from first shot to start of bleeding was observed in 24% of 21 cases (n = 5; stun-to-stick interval 65, 69, 73, 75 and 76 s, respective). Two of these reindeer (69 and 76-s stun-to-stick interval) were stunned by medium experienced and the remaining three reindeer by the shortest experienced stunning operator, respectively). When deemed necessary, the reindeer were re-stunned with the same CBG and by the same operator.

### Ineffective versus effective stuns and indicators at ineffective stuns

Ineffective stuns were observed in 1.3% of cases (21/1,590; 5 calves and 16 adults). These cases were observed equally by both the stunning operator and the observer and mainly involved adult male reindeer (11/21, 52.4%) and during the winter season (18/21). During the autumn three adult males were re-stunned, and no cases occurred during the spring slaughter of reindeer being fed in corrals (0/130). The number of ineffective (i.e. re-stunned) stuns versus effective stuns for calves and adults as well as between male and female reindeer showed a statistically significant difference (P < 0.001, P < 0.05; respectively). However, there was no significant difference of ineffective stuns between years of experience of the stunning operator, between reindeer from forest versus mountain origin or between slaughter seasons. The results structured as a 2 × 2 matrix of frequencies can be seen in supplemental material (S2).

The most frequently recorded indicator for ineffective stunning was the reindeer remaining in a standing posture following the incorrect placing of the CBG shot, mainly for adult males. In two cases, the stunned reindeer (one calf and one adult female) displayed spontaneous blinking, and no other signs of consciousness were observed (Table 5). All these 21 cases were observed and documented with clearly seen incorrect CBG placement on the skull of reindeer. Observations on indicators made by the stunning operator and the observer were the same at every stun.

**Table 5:** Reindeer with ineffective stuns, with more than one shot after indicators noted. Details*

Abattoir	Extra action done	Indicator seen	Type of animal	Grazing area	Type of stunning	Level of experience**
AA	Two shots	Standing posture	Adult male	Mountain	Cartridge-powered	Long
AA	Two shots	Standing posture	Adult male	Mountain	Cartridge-powered	Long
AB	Two shots	Standing posture	Calf	Forest	Pneumatic	Long
AB	Two shots	Standing posture	Calf	Forest	Pneumatic	Long
AB	Two shots	Standing posture	Adult male	Forest	Pneumatic	Long
AB	Two shots	Standing posture	Adult male	Forest	Pneumatic	Long
AB	Two shots	Standing posture	Adult female	Forest	Pneumatic	Long
AA	Three shots, bled in the stunning box	Standing posture	Adult male	Mountain	Cartridge-powered*	Long
AA	Two shots	Standing posture	Adult male	Mountain	Pneumatic	Medium
AA	Two shots	Standing posture	Calf	Mountain	Pneumatic	Medium
AB	Two shots	Standing posture	Adult female	Forest	Pneumatic	Medium
AB	Two shots	Standing posture	Adult female	Forest	Pneumatic	Medium
AB	Two shots	Standing posture	Adult male	Forest	Pneumatic	Medium
AB	Two shots	Standing posture	Adult male	Mountain	Pneumatic	Medium
AB	Two shots	Standing posture	Calf	Forest	Pneumatic	Short
AB	Two shots	Standing posture, eye blinking	Calf	Mountain	Pneumatic	Short
AB	Two shots	Standing posture	Adult male	Mountain	Pneumatic	Short
AB	Three shots	Standing posture	Adult female	Mountain	Pneumatic	Short
AB	Three shots	Standing posture	Adult male	Forest	Pneumatic	Short
AB	Three shots	Standing posture	Adult male	Forest	Pneumatic	Short
AB	Three shots	Standing posture, eye blinking	Adult female	Forest	Pneumatic	Short

*The abattoir (AA, AB), extra action taken by the stunning operator, indicators noted by the stunning operator and the observer, type of animal stunned, type of stunning used and level of experience of the stunning operator. Stun-to-stick interval in seconds was documented from the last shot. Back-up cartridge-powered captive bolt gun used, support person loading for the third shot, stacked by the stunning operator

**Level of experience of stunning operator in years: Five = long, Three = medium, One year = short experience

## Discussion

The vast majority of the reindeer in our study were effectively stunned even if the 60 s limit between stunning and start of the exsanguination exceeded in some cases. The mean stun-to-stick interval for effective stuns were shorter as well as the proportions of delayed stickings for the more experienced stunning operators compared to the less experienced operators. The mean stun-to-stick interval was shorter at abattoir AB compared to abattoir AA, but the proportions of reindeer with delayed stickings did not differ. Adult reindeer as bigger and heavier animals had longer stun-to-stick interval than calves that are easier to hoist before sticking. The design of the stunning box and type of the CBG was the same in the two abattoirs. The slower hydraulic hoisting system at the abattoir AA was affecting the stun-to-stick time in general even if the limit of 60 s were not compromised more often compared to abattoir AB. This is a good example of the importance of good design and active maintenance of the equipment along the slaughter line.

### Effective stuns

In all cases with effective stuns, the reindeer in our study did not show any indicators of consciousness but did display relevant signs of unconsciousness and were considered unconscious during the entire stun-stick period, i.e. up to 103 s following effective stunning, and remained unconscious until bleeding was completed. Reindeer were considered dead when exhibiting a complete absence of any signs of life, such as breathing, brainstem reflexes and heartbeats, in combination with sufficient bleeding for a long enough period of time [19]. Much shorter time, maximum 54 s stun-to-stick interval, was required to avoid any risk of animals regaining consciousness during the slaughter process with head-only electrical stunning of red deer (*Cervus elaphus*) [34]. Using a puntilla knife to lacerate the central nervous tissue and spinal cord, e.g. a hook-bladed knife or similar instrument introduced via the back of the neck, has been illegal for reindeer in Sweden since 1982 [35] because the method does not result in a reliable stun, although it may immobilize the animal [36]. Occipital captive-bolt stunning has been used for deer in the British Isles since the 1980s [37] but is not approved for any species in Sweden, as an occipital shot may harm central nervous system functions in a way that causes loss of posture, respiratory arrest and absence of corneal reflexes, while leaving the animal fully conscious but paralysed. The penetrative captive bolt has been shown to be more effective as a stunning method than the head-only electrical stunning with induced epileptiform brain status [38]. Electrical or gas stunning of reindeer has never been practiced in Sweden and is prohibited [9] due to insufficient data to prove efficacy in this species. In our study, frontally positioned CBG stunning resulted in a good stun quality lasting for more than twice the time period seen in the older red deer study [34].

For reindeer and other farmed game animals, degree of tameness can be an important factor to consider during handling [39]. There is a general assumption among reindeer owners and slaughters that reindeer from Sami villages located in forest areas all year round are more accustomed to human presence, and hence tamer, as well as reindeer fed in corrals near the herders for different reasons and slaughtered late spring, than reindeer from mountain areas. In our study we could not see any correlation between ineffective stunning and reindeer with different geographical origin. Anyhow, none of the reindeer slaughtered after being fed in corrals in the spring were stunned ineffectively. The sample size is too low to show any statistical significance. Moreover, the corral fed reindeer in the spring had shorter mean stun-to-stick interval than reindeer at winter and autumn slaughter as well as the reindeer from mountain pasture had shorter mean stun-to-stick interval compared to forest reindeer. The reason for these results should be studied more closely in future.

### Ineffective stuns

The ineffective stuns resulting in re-stunning in our study were caused by an incorrect CBG placement of the first or second shot, usually because of the reindeer not standing still or suddenly throwing its head. The category of animals most often stunned ineffectively were adult males, and female reindeer were more often stunned ineffectively than calves. Re-stunning mainly took place during the winter, a minor proportion in autumn and no cases occurred in the spring. Neither the level of experience of the stunning operator nor origin of reindeer was significantly related to the incidence of re-stunning.

In stunning reindeer with a CBG, the shot is delivered to the front, at the intersection of the sagittal line and horizontal line between the upper lines of the eyes or, if the antlers prevent this positioning, behind the antlers in the occipital position, in accordance with Swedish guidelines [9]. The positioning of the CBG can be difficult if the head is not well restrained, especially if antlers make access to the correct positioning of the gun more complicated. Moreover, when individual reindeer are less tame, they are more difficult to handle in the stun box. The stunning operator must wait until the animal is momentarily standing still or, in some cases, manually restrain the animal’s head by seizing the (non-velvet) antlers. The incidence of ineffective stuns among reindeer from mountain areas was not higher than that among reindeer from forest areas.

The stunner operator must strive for the optimal position (Fig. 2) of the shot to optimize an effective stunning result. Even a suboptimal orientation of the bolt and a CBG shot in tangent to the optimal point can still result in an effective stun if a vast and multiple damages of the brain is caused [19]. Despite that the SOP recommendation is very important, as deviations in the stun position can result in ineffective stuns, as clearly seen in our study and demonstrated for water buffalo [40] and for cattle [41]. Reindeer are smaller animals than those species (calves approximately 30–50 kg, adult females 60–90 kg, males 100–150 kg) [14], with a smaller cranium and ditto brain making it even more important to hit the correct target point. Alpacas (*Vicugna pacos*), a species with a small cranium, are reported to be stunned effectively by a CBG, although in some cases double shots are required to achieve effective stunning [42].

Ineffectively stunned animals constitute a serious animal welfare issue; hence, careful observation of indicators is required to determine that the animals are effectively stunned. Effectively stunned reindeer exhibit loss of posture, absence of attempts to regain posture, absence of rhythmic breathing, eyeballs in a fixed forward stare (no movement, no eye reflexes), dilated pupils, limited kicking and minimal reaction to the sticking procedure. Possible indicators indicating an ineffective stun in reindeer and other mammalian species include retaining or regaining posture, display of corneal reflex or other eye reflexes, blinking, breathing, any combination of eyeball rotations, eyeball twitching, nostril flaring, groaning or severe righting reflex [20, 21, 23, 43, 44]. Multiple indicators should be used to determine unconsciousness during the whole stunning and bleeding process, until the animal is dead [23]. Mechanical stunning (CBG) results in pathophysical damage to the brain tissue and affects the eye reflexes, such as loss of corneal reflex. Loss of posture and rhythmic breathing are considered as behavioural indicators. Reflex-based physiological indicators, which are automatic stereotyped movements produced as the direct result of a stimulus, include e.g. the corneal reflex [45]. The multiple indicators used in this study were relevant for mechanical stunning and observed from first shot in the stunning box until the reindeer was bled, whereafter the abattoir staff were instructed to continue intermediate monitoring until the animals were dead, as usual, albeit outside of this study.

### Future studies

A further analysis of the interacting factors in stunning reindeer should be supported by a larger data set emanating from several abattoirs, a larger number of reindeer from mountain and forest areas during different time of the slaughter seasons and stunned by several stunning operators with different experienced time of stunning. To optimize the SOP as well as design and maintenance of the stunning box and other equipment at every abattoir approved and used for reindeer slaughter is needed. This can be done after the level of importance of the different variables on the effective stuns in reindeer slaughter is defined. The analysis should preferably be done by statistical modelling.

Slaughtering and handling animals that live under feral conditions is more complex than slaughtering animals that are accustomed to and handled by humans. Reindeer, i.e. more or less non-domesticated animals that are taken into an unusual and for them unsustainable situation such as a slaughterhouse and its surroundings, place different demands on the staff's animal welfare knowledge, about animals that live their lives more or less in the wild. Research on the perceptual and cognitive abilities of animals living under feral conditions is strikingly limited, there is a gap between what is known and what we want to achieve to avoid mistreatment that causes inappropriate measures. Knowledge of how animals living under feral conditions perceive and interact with their environment is needed, and the animal welfare for these animals in the slaughterhouse is particularly important because all steps in the process, from unloading, housing and handling to stunning and slaughter itself, offer the potential for distress and suffering.

## Conclusions

The level of experience of the stunning operator, the type of animal as well as the design and maintenance of the stunning equipment are of importance in achieving an effective stun of reindeer.

Stun-to-stick interval below 60 s is reachable with current facilities when correctly applying SOPs at reindeer abattoirs. Even with a delayed stun-to-stick interval, i.e. exceeding 60 s, an effective stun quality was shown to last until bleed-out. The most frequently recorded indicator for ineffective stunning was the reindeer remaining in a standing posture following the incorrect placing of the CBG shot, mainly for adult males.

To enhance animal welfare at slaughter, a supervised training period for operators included in the course for certificate of competence, continuous development of SOP: s and maintenance of the stunning equipment are needed. The effective official controls should focus on these most important factors.

## Supplementary Information

Supplementary Material 1.

## Data Availability

The datasets used during the current study can be requested from the Swedish Food Agency.

## Abbreviations

CA: Competent Authority
CBG: Captive Bolt Gun
CCA: Central Competent Authority
EFSA: European Food Safety Authority
EU: European Union
FBO: Food Business Operator
OV: Official Veterinarian
SBA: Swedish Board of Agriculture
SFA: Swedish Food Agency
SOP: Standard Operating Procedures
WOAH: World Organization for Animal Health

## Declarations

### Prior publication

Data have not been published previously.
