# Supplementary material for: Factors affecting the effectiveness of captive-bolt stunning of reindeer (Rangifer tarandus tarandus L.) at commercial slaughter in Sweden
Source: Acta Vet Scand. 2026 Jan 28;68:12. doi: 10.1186/s13028-026-00852-x (PMC12924502; doi:10.1186/s13028-026-00852-x)
Supplement: Supplementary file 1 — Supplementary Material 1. [file 13028_2026_852_MOESM1_ESM.docx]

Supplemental material – Tables S1 and S2

Table S1. Number of reindeer of effective stuns with stun-to-stick interval >60 seconds versus ≤ 60 seconds. Details*.

| Stun-to-stick interval | Long experience | Medium experience | In total | P- value |
| --- | --- | --- | --- | --- |
| >60 sec | 3 | 48 | 51 |  |
| ≤60 sec | 695 | 617 | 1312 |  |
| In total | 698 | 665 | 1363 | P < 0.001 |
|  |  |  |  |  |
| Stun-to-stick interval | Medium experience | Short experience | In total |  |
| >60 sec | 48 | 32 | 80 |  |
| ≤60 sec | 617 | 174 | 791 |  |
| In total | 665 | 206 | 871 | P = 0.008 |
|  |  |  |  |  |
| Stun-to-stick interval | Calf | Adult | In total | P-value |
| >60 sec | 40 | 43 | 83 |  |
| ≤60 sec | 930 | 556 | 1486 |  |
| In total | 970 | 599 | 1569 | P = 0.14 |
| Stun-to-stick interval | Male | Female | In total | P -value |
| >60 sec | 13 | 30 | 43 |  |
| ≤60 sec | 212 | 344 | 556 |  |
| In total | 225 | 374 | 599 | P = 0.90 |
| Stun-to-stick interval | Winter | Autumn | In total | P-value |
| >60 sec | 80 | 3 | 83 |  |
| ≤60 sec | 1246 | 110 | 1356 |  |
| In total | 1326 | 113 | 1439 | P = 0.20 |
|  |  |  |  |  |
| Stun-to-stick interval | Abattoir AA | Abattoir  AB | In total | P-value |
| >60 sec | 40 | 43 | 83 |  |
| ≤60 sec | 545 | 941 | 1486 |  |
| In total | 585 | 984 | 1569 | P = 0.37 |
|  |  |  |  |  |
| Stun-to-stick interval | Cartridge | Pneumatic | In total | Chi-squared, p-value |
| >60 sec | 7 | 76 | 83 |  |
| ≤60 sec | 198 | 1288 | 1486 |  |
| In total | 205 | 1364 | 1569 | P = 0.80 |
|  |  |  |  |  |
| Stun-to-stick interval | Forest | Mountain | In total | Chi-squared, p-value |
| >60 sec | 33 | 50 | 83 |  |
| ≤60 sec | 543 | 943 | 1486 |  |
| In total | 576 | 993 | 1569 | P = 1 |

* In relation to level of experience of the stunning operator, calf versus adult, male versus female, winter versus autumn, abattoir AA versus AB, cartridge versus pneumatic gun, and forest versus mountain origin. Spring slaughter was excluded because of zero cases exceeding 60 seconds. P- value counted by McNemar test and in cases with five or less observations per cell with Fisher Exact test.

Table S2. Number of stunned reindeer and stunning result (ineffective versus effective) after the first shot in relation to calf versus adult reindeer, male versus female, level of experience of the stunning operator, forest versus mountain origin, spring versus Autumn and Winter, Winter versus Autumn.

| Animal type | Stunning quality | | | P -value |
| --- | --- | --- | --- | --- |
|  | Ineffective | Effective | In total |  |
| Calf | 5 | 970 | 975 |  |
| Adult | 16 | 599 | 615 |  |
| In total | 21 | 1569 | 1590 | P < 0.001 |
|  |  |  |  |  |
| Male | 11 | 225 | 236 |  |
| Female | 5 | 374 | 379 |  |
| In total | 16 | 599 | 615 | P = 0.02 |

.

| Level of experience of stunning operators | Stunning quality | |  | P-value |
| --- | --- | --- | --- | --- |
|  | Ineffective | Effective | In total |  |
| Long | 8 | 698 | 706 |  |
| Medium | 6 | 665 | 671 |  |
| In total | 706 | 1363 | 1377 | P = 1 |
|  |  |  |  |  |
| Medium | 6 | 665 | 671 |  |
| Short | 7 | 206 | 213 |  |
| In total | 13 | 871 | 884 | P = 0.17 |

| Origin of reindeer | Stunning quality | |  | P-value |
| --- | --- | --- | --- | --- |
|  | Ineffective | Effective | In total |  |
| Forest | 12 | 576 | 588 |  |
| Mountain | 9 | 993 | 1002 |  |
| In total | 21 | 1569 | 1590 | P = 0.43 |
| Slaughter season | Stunning quality | |  | P-value |
|  | Ineffective | Effective | In total |  |
| Spring | 0 | 130 | 130 |  |
| Winter and Autumn | 21 | 1439 | 1460 |  |
| In total | 21 | 1569 | 1590 | P = 0.89 |
|  |  |  |  |  |
| Winter | 18 | 1327 | 1345 |  |
| Autumn | 3 | 112 | 115 |  |
| In total | 21 | 1439 | 1460 | P = 0.81 |
